# Prostatic fossa calculus

**DOI:** 10.11604/pamj.2015.21.236.7114

**Published:** 2015-07-31

**Authors:** Yassine El Abiad, Mohammed Alami

**Affiliations:** 1 Urology Department, My Ismail Military Hospital, Meknès, Morocco

**Keywords:** Calculus, prostatic fossa, endoscopy

## Image in medicine

A 70-year-old man presented for evaluation of recurrent urinary tract infection. He had history of hypertension and had undergone suprapubic prostatectomy 8 years ago for enlarged obstructive prostate. Digital rectal examination found a stony irregular prostate and urine culture grew Escherichia Coli that was susceptible to Cephalosporines. As part of the evaluation, a plain radiograph was performed and incidentally showed a radiopaque prostatic calculus (Red arrows). A retrograde urethrocystography, performed after a 10-days course of antibiotics, confirmed the presence of an approximately 35 mm non-obstructive calculus occupying almost the whole prostatic bed (Red arrows) with a stricture of the membranous urethra (Yellow arrow), the bladder however showed no abnormalities (Orange arrows). Stones that form in the prostatic fossa are uncommon but can occur after all forms of prostatectomy. They are caused by a ligature, infection, a chip of prostate left behind in the prostatic bed or as a result of an iatrogenic urethral stricture. A meticulous operative and postoperative care is the best prevention of such a complication. Our patient was treated endoscopically by the combination of urethrotomy and transurethral ultrasonic lithotripsy. At 6 months follow-up, he voided properly and had a sterile urine culture.

**Figure 1:**
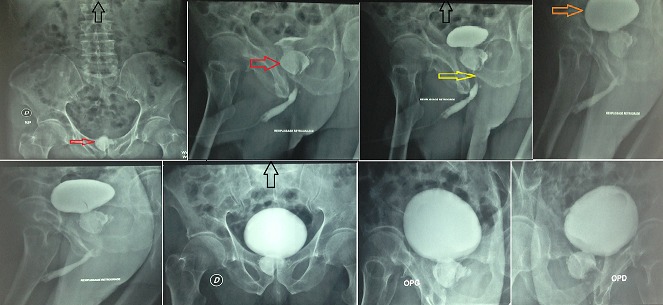
Retrograde urethrocystography showing a non obstructive prostatic calculuc

